# O071. The association between meteoropathy, depression, hopelessness and quality of life in medication-overuse headache patients

**DOI:** 10.1186/1129-2377-16-S1-A50

**Published:** 2015-09-28

**Authors:** Samantha Bellini, Monica Migliorati, Federica Ricci, Denise Erbuto, Maurizio Pompili

**Affiliations:** Department of Neurosciences Suicide Prevention Centre, Sapienza University of Rome, Rome, Italy

## Background

Medication-overuse headache (MOH) is one of the most common forms of chronic daily headache (CDH)[1], with a prevalence in the adult general population of 1%-2%[2]. MOH is often comorbid with emotional disturbances and disordered personality traits. The aim of the present study was to determine whether depression, hopelessness and quality of life were associated with meteoropathy in MOH patients. The most frequent symptom of meteoropathy is the exacerbation of acute or chronic forms of pain in many parts of the body, in and of itself inflamed and/or degenerated.

## Materials and methods

Participants were 203 consecutive adult outpatients, of which 165 females (81.3%), admitted to the local Headache Centre of the Sant'Andrea Hospital in Rome, Italy. Inclusion criteria were a diagnosis of MOH, and an age of 18 years or older. Exclusion criteria were comorbidity with major disorders of the central nervous system, delirium and/or any condition affecting the patient's ability to complete the assessment. The average age of participants was 46.99±11.99. Patients participated voluntarily in the study, and each subject provided written informed consent. Patients were administered the BDI II, BHS, Q-LES-Q and the METEO-Q.

## Results

The results showed middle-high levels of meteoropathy in our sample. Men showed higher level of meteoropathy than female. The intensity of meteoropathy correlates significantly with the levels of depression (r = .253; p < 0.01), hopelessness (r = .151; p < 0.05), and with some subscales of the Q-LES-Q (-.282<r>-.105; p < 0.01). The quantitative level of meteoropathy correlates with the levels of depression (r = .376; p < 0.01), but not with the levels of hopelessness. The METEO-Q was significantly associated with any dimensions of the Q-LES-Q when controlling for the presence of other variables.

## Conclusions

Our data seem to confirm that patients with MOH are prone to experiment the high levels of meteoropathy both in quality and in intensity, confirming that MOH has a negative impact on quality of life. Moreover, meteoropathy was found to be associated with levels of depression, and with physical health, emotions, social relations, and general activities. Possibly neurophysiological and endocrinological alterations linked to climatic changes play a role in affecting quality of life. An integrated approach that includes the neurological and psychological fields may be particularly useful, because of the bi-directionality of the migraine-depression association, of the crucial role of depression in the transformation of migraine in MOH, and could minimize the risk of chronic headaches, improving the prognosis.

Written informed consent to publication was obtained from the patient(s).

## Conflict of Interest

The authors report no conflicts of interest.
